# miRNA-Seq Tissue Diagnostic Signature: A Novel Model for NSCLC Subtyping

**DOI:** 10.3390/ijms241713318

**Published:** 2023-08-28

**Authors:** Radoslaw Charkiewicz, Anetta Sulewska, Alicja Charkiewicz, Attila Gyenesei, Bence Galik, Rodryg Ramlau, Cezary Piwkowski, Rafal Stec, Przemyslaw Biecek, Piotr Karabowicz, Anna Michalska-Falkowska, Wojciech Miltyk, Jacek Niklinski

**Affiliations:** 1 Center of Experimental Medicine, Medical University of Bialystok, 15-369 Bialystok, Poland; 2 Department of Clinical Molecular Biology, Medical University of Bialystok, 15-269 Bialystok, Poland; anetta.sulewska@umb.edu.pl; 3 Department of Analysis and Bioanalysis of Medicines, Medical University of Bialystok, 15-089 Bialystok, Poland; alicja.charkiewicz@umb.edu.pl (A.C.); wojciech.miltyk@umb.edu.pl (W.M.); 4 Szentagothai Research Center, Genomic and Bioinformatic Core Facility, H-7624 Pecs, Hungary; gyenesei.attila@pte.hu (A.G.); galik.bence@pte.hu (B.G.); 5 Department of Oncology, Poznan University of Medical Sciences, 60-569 Poznan, Poland; rodrygramlau@ump.edu.pl; 6 Department of Thoracic Surgery, Poznan University of Medical Sciences, 60-569 Poznan, Poland; cpiwkow@ump.edu.pl; 7 Department of Oncology, Medical University of Warsaw, 02-091 Warsaw, Poland; rafal.stec@uckwum.edu.pl; 8 Faculty of Mathematics and Information Science, Warsaw University of Technology, 00-662 Warsaw, Poland; przemyslaw.biecek@pw.edu.pl; 9 Biobank, Medical University of Bialystok, 15-269 Bialystok, Poland; piotr.karabowicz@umb.edu.pl (P.K.); anna.michalska-falkowska@umb.edu.pl (A.M.-F.)

**Keywords:** miRNA signature, NSCLC subtyping, molecular targeted therapy, NGS

## Abstract

Non-small cell lung cancer (NSCLC) encompasses distinct histopathological subtypes, namely adenocarcinoma (AC) and squamous cell lung carcinoma (SCC), which require precise differentiation for effective treatment strategies. In this study, we present a novel molecular diagnostic model that integrates tissue-specific expression profiles of microRNAs (miRNAs) obtained through next-generation sequencing (NGS) to discriminate between AC and SCC subtypes of NSCLC. This approach offers a more comprehensive and precise molecular characterization compared to conventional methods such as histopathology or immunohistochemistry. Firstly, we identified 31 miRNAs with significant differential expression between AC and SCC cases. Subsequently, we constructed a 17-miRNA signature through rigorous multistep analyses, including LASSO/elastic net regression. The signature includes both upregulated miRNAs (hsa-miR-326, hsa-miR-450a-5p, hsa-miR-1287-5p, hsa-miR-556-5p, hsa-miR-542-3p, hsa-miR-30b-5p, hsa-miR-4728-3p, hsa-miR-450a-1-3p, hsa-miR-375, hsa-miR-147b, hsa-miR-7705, and hsa-miR-653-3p) and downregulated miRNAs (hsa-miR-944, hsa-miR-205-5p, hsa-miR-205-3p, hsa-miR-149-5p, and hsa-miR-6510-3p). To assess the discriminative capability of the 17-miRNA signature, we performed receiver operating characteristic (ROC) curve analysis, which demonstrated an impressive area under the curve (AUC) value of 0.994. Our findings highlight the exceptional diagnostic performance of the miRNA signature as a stratifying biomarker for distinguishing between AC and SCC subtypes in lung cancer. The developed molecular diagnostic model holds promise for providing a more accurate and comprehensive molecular characterization of NSCLC, thereby guiding personalized treatment decisions and improving clinical management and prognosis for patients.

## 1. Introduction

Non-small cell lung cancer (NSCLC) is a complex and heterogeneous disease with a dynamic and intricate genetic architecture characterized by diverse molecular alterations that contribute to tumor development and drug resistance [1]. In addition to genetic mutations, epigenetic modifications play a crucial role in NSCLC development and progression. The epigenetic landscape of NSCLC is characterized by various alterations, including DNA methylation, histone modifications, and non-coding RNA expression [2,3]. These changes can affect the expression of key protein-coding genes involved in cellular processes such as cell cycle regulation, apoptosis, invasion, metastasis, angiogenesis, immune response, and drug resistance [3]. Epigenetic alterations can also interact with genetic mutations and modulate their effects on tumor phenotype and behavior [3].

MicroRNAs (miRNAs) are small non-coding RNAs that regulate gene expression by degrading or repressing mRNA targets. Depending on their target genes and cellular context, miRNAs can act as either oncogenes or tumor suppressors. Altered expression of miRNAs has been associated with lung cancer initiation, progression, metastasis, drug resistance, and prognosis. MiRNAs also have potential as biomarkers for NSCLC diagnosis, subtype classification, prediction of treatment response, and prognosis [3,4,5,6,7].

The field of cancer genomics has been transformed by the advent of next-generation sequencing (NGS), which allows for rapid and cost-effective sequencing of large portions of the genome or transcriptome and detection of a variety of genomic alterations, including point mutations, insertions/deletions, copy number variations, and gene fusions. NGS offers a comprehensive and unbiased profiling of the tumor genome, as well as the ability to identify novel or rare mutations of clinical relevance [8,9,10]. In addition, NGS can identify epigenetic alterations, such as DNA methylation and non-coding RNA expression [11]. Investigating novel genetic and epigenetic biomarkers utilizing high-throughput technologies represents a significant advancement in distinguishing squamous cell lung carcinoma (SCC) and adenocarcinoma (AC) compared to traditional histopathological examination. While histopathological diagnosis remains the established benchmark, it is not without its well-known challenges and constraints. Immunohistochemical staining (IHC) of tumors offers only partial enhancement of classification accuracy due to varying sensitivities/specificities of individual markers, technical discrepancies leading to staining reaction variability, tumor heterogeneity, and the absence of standardized quantitative interpretation of staining results. Furthermore, the mutability of histological subclassifications of NSCLC can be associated with sampling and assessment procedures [6]. It is estimated that in approximately 15–20% of cases, precise NSCLC subtype determination using classical histopathological techniques is unattainable [12]. In such instances, cancer is identified as non-small cell carcinoma without subtype specification, referred to as NOS (not otherwise specified).

The rapidly evolving landscape of personalized medicine and the increasing significance of emerging anti-cancer strategies underscore the imperative for accurate histopathological categorization of NSCLC, accompanied by thorough assessment of somatic mutational profiles within pulmonary neoplasms [13,14,15]. Targeted therapeutic interventions yield optimal clinical utility predominantly within the domain of non-squamous NSCLC variants [15,16]. Epidermal growth factor receptor (EGFR) tyrosine kinase inhibitors (e.g., erlotinib, gefitinib, and afatinib), employed as first-line treatments for advanced NSCLC, demonstrate pronounced efficacy primarily against histotypes other than squamous carcinoma [17]. The integration of monoclonal anti-VEGF antibodies (e.g., bevacizumab) with conventional cytotoxic regimens is judicious for patients devoid of life-threatening hemorrhagic propensities, a characterization inclusive of those exempt from a diagnosis of squamous cell carcinoma [18]. Moreover, the therapeutic dividend attributed to anaplastic lymphoma kinase (ALK) inhibitors (e.g., crizotinib, ceritinib, alectinib, and brigatinib) is confined to individuals harboring lung adenocarcinomas bearing the ALK fusions [19]. Singularly endorsed by the FDA for molecularly targeted intervention in advanced squamous cell carcinoma is necitumumab, in synergy with gemcitabine and cisplatin, administered as a front-line therapeutic regimen [20]. The utilization of monoclonal IgG1 antibodies targeting EGFR does not find applicability in the context of non-squamous histotypes [20]. Immunocompetent agents such as pembrolizumab, when employed concomitantly with chemotherapeutic protocols, afford a therapeutic avenue for patients lacking a definitive diagnosis of squamous subtype [21]. The ADAURA clinical study, probing the efficacy of adjuvant osimertinib therapy, has unveiled notable amelioration in disease-free survival for early and locally advanced non-small cell lung carcinoma patients bearing EGFR mutations [22].

Evidently, the utmost importance of both targeted and immunological therapeutic approaches in clinical practice highlights the compelling need for an enhanced and more meticulous histopathological classification of NSCLC. This classification stands as a fundamental factor in precisely stratifying patients for personalized therapeutic strategies.

Previous studies conducted by our research team utilizing microarrays and quantitative polymerase chain reaction (qPCR) have shown promising results with miRNA and long non-coding RNA (lncRNA) signatures [23,24]. Building upon this existing foundation, the primary objective of this study is to establish a novel molecular diagnostic model that effectively discriminates between the two primary histopathological subtypes of non-small cell lung cancer (NSCLC), namely adenocarcinoma (AC) and squamous cell lung carcinoma (SCC), utilizing the tissue-specific expression profiles of miRNAs.

The model integrates analytical and bioinformatic techniques to generate a miRnome tissue profile that accurately stratifies NSCLC subtypes. This approach has the potential to provide a more precise and comprehensive molecular characterization of NSCLC than conventional methods, such as histopathology or immunohistochemistry. It could also improve the clinical management and prognosis of NSCLC patients by guiding personalized treatment decisions based on miRNA expression profiles.

## 2. Results

### 2.1. Differentially Expressed miRNA to Differentiate AC from SCC

The preprocessed data was subjected to filtering in order to identify differentially expressed miRNAs (DE miRNAs) based on their statistical significance and expression level differences. Robust evidence of differential expression was obtained through fold change analysis utilizing the Limma package version 3.32.0, with modified *t*-tests and false discovery rate (FDR) *p*-values.

**Figure 1:**
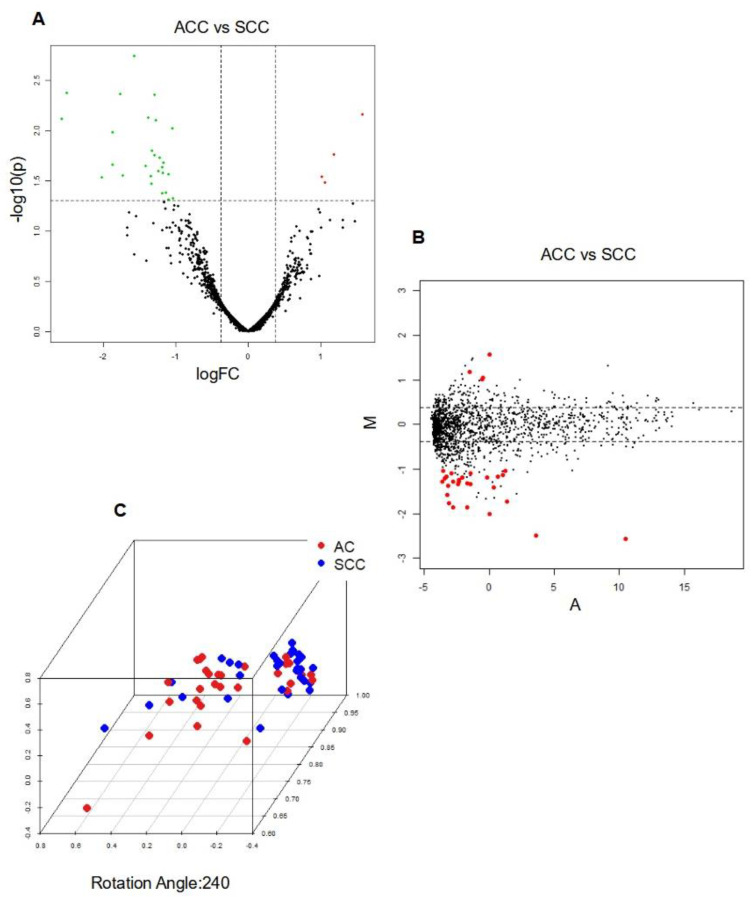
Integrative analysis of differentially expressed miRNAs in AC vs. SCC (**A**) Volcano Plot; Legend: The *y*−axis represents the logarithm (base 10) of the *p*−values, while the *x*−axis represents the logarithm (base 2) of the fold change calculated for the comparison group versus the baseline group. The plot visualizes the relationship between the statistical significance (*p*−values) and the magnitude of the fold change for the measured characteristics. Dashed lines indicate the filtering thresholds used, with upregulated genes depicted in red and downregulated genes in green; (**B**) MDA Plot; Legend: Positive values indicate higher expression in AC, while negative values indicate higher expression in SCC; and (**C**) PCA Plot; Legend: Sample Separation: Distinct clustering of AC and SCC samples indicates differential miRNA expression profiles between the two cancer types; Sample Similarity: Proximity on the plot reflects similarity in miRNA expression patterns; Contribution of Principal Components: Axes (PCs) represent miRNA expression combinations. The contribution of each PC indicates key miRNAs or patterns driving AC vs. SCC separation.

Among the 31 DE miRNAs identified, 27 miRNAs exhibited downregulation in adenocarcinoma (AC) compared to squamous cell carcinoma (SCC), whereas only 4 miRNAs (hsa-miR-3617-5p, hsa-miR-4709-5p, hsa-miR-1294, and hsa-miR-4636) were found to be upregulated in AC compared to SCC. The top 5 downregulated miRNAs, ranked from 1 to 5, were hsa-miR-944, hsa-miR-205-5p, hsa-miR-383-5p, hsa-miR-3927-3p, and hsa-miR-448. The results, including the identified DE miRNAs and their corresponding expression changes, are presented in Table 1. Moreover, Figure 1 provides visual representation of these results, aiding in the interpretation of the observed expression patterns.

### 2.2. miRNA Signature to Differentiate AC from SCC

**Figure 2:**
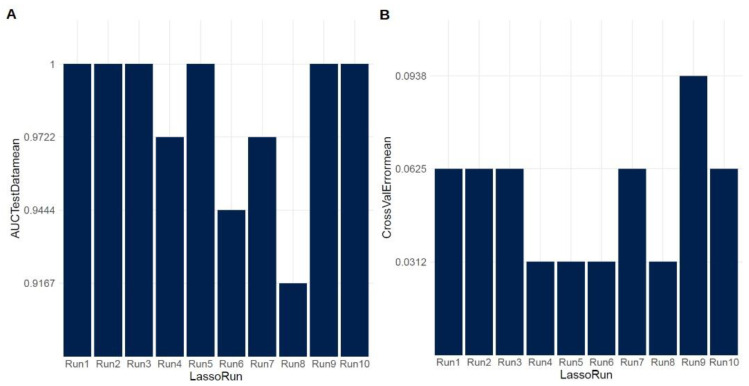
LASSO regression analysis results (**A**) for the test data and (**B**) for cross-validation.

**Figure 3:**
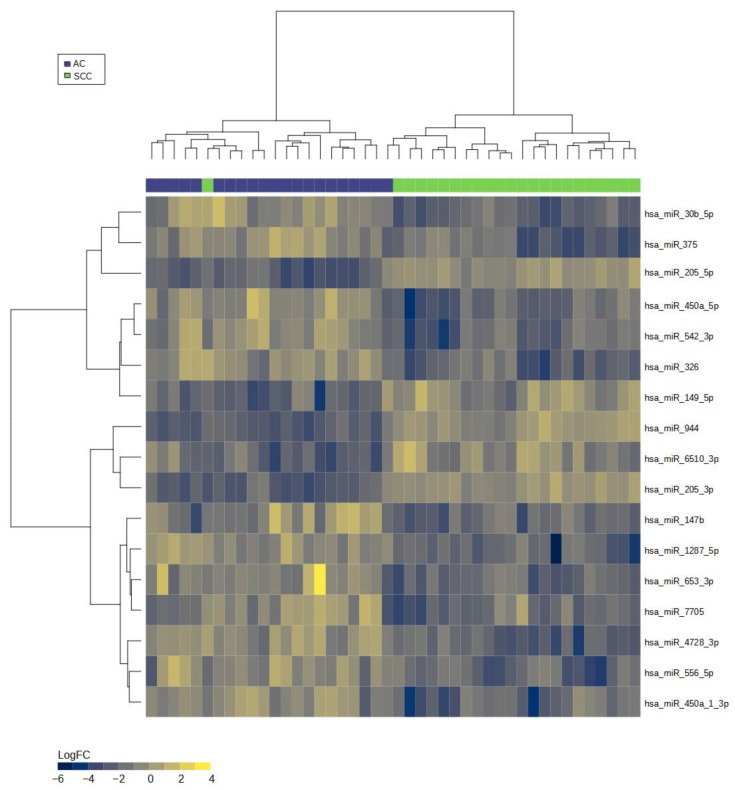
Heatmap of miRNA expression signature for distinguishing adenocarcinoma (AC) and squamous cell carcinoma (SCC) groups. Legend: The heatmap visualizes the expression patterns of miRNAs, with rows representing individual miRNAs and columns representing samples. The color scale represents the expression levels, where higher expression is indicated by warmer colors (yellow) and lower expression by cooler colors (blue). The distinct patterns of miRNA expression in AC and SCC samples can be observed, indicating their potential as biomarkers for discriminating between the two cancer types.

Applying an alternative statistical approach involving multiple stages, including differential abundance analysis, LASSO/elastic-net regression, log2 transformation, and MDS plot generation, we identified a robust miRNA-seq signature comprising 17 miRNAs, which effectively discriminate NSCLC subtypes. Within this set, 12 miRNAs demonstrated upregulation, while 5 miRNAs exhibited downregulation in AC compared to SCC. Notably, we observed that four downregulated miRNAs (hsa-miR-944, hsa-miR-205-5p, hsa-miR-205-3p, and hsa-miR-6510-3p) were common to both the DE analysis and the developed signature. Table 2 presents the significant miRNAs that effectively discriminate AC from SCC, while Figure 2 illustrates the results of the LASSO regression in both the test and cross-validation data, respectively. To visualize the miRNA signature that distinguishes AC from SCC lung carcinomas, we generated a heatmap (Figure 3).

### 2.3. Evaluation of the Diagnostic Utility of the Identified miRNA Signature

**Figure 4:**
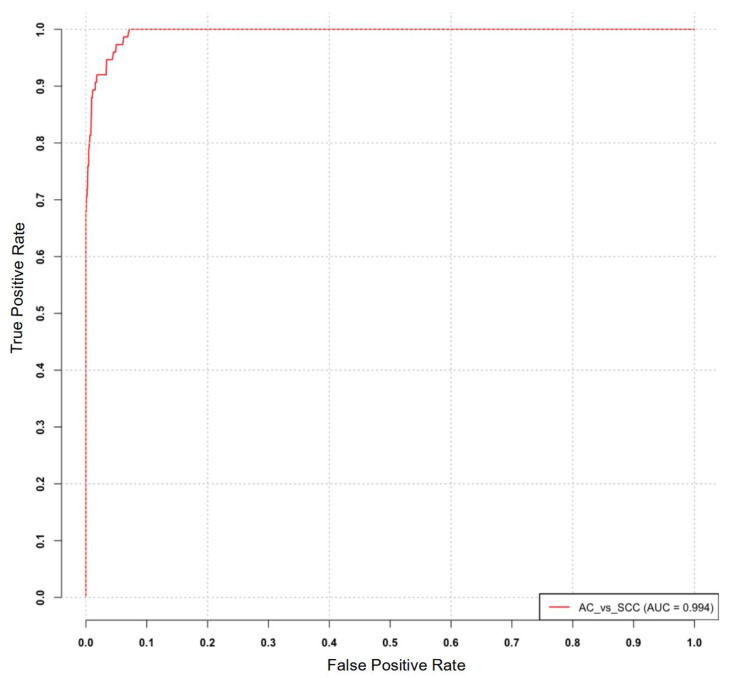
ROC curve analysis for the identified miRNA signature in distinguishing adenocarcinoma (AC) and squamous cell carcinoma (SCC).

The diagnostic utility of the identified miRNA signature was evaluated by constructing a receiver operating characteristic (ROC) curve, which plots the true positive rate (TPR) against the false positive rate (FPR) across different threshold settings. The ROC curve was generated using the MetaseqR Bioconductor package, which incorporated a matrix of *p*-values derived from the previous analysis, a ground truth vector for differential expression, and a specified significance level. The ROC analysis demonstrated an impressive area under the curve (AUC) value of 0.994, indicating the strong diagnostic potential of the identified miRNA signature. Figure 4 illustrates the ROC curve depicting the performance of the miRNA signature in diagnosis.

## 3. Discussion

Lung cancer is a complex disease with distinct heterogeneity, encompassing two primary histological subtypes: adenocarcinoma (AC) and squamous cell carcinoma (SCC) [25,26]. These subtypes exhibit divergent molecular characteristics, clinical features, prognosis, and therapeutic responses [25,26]. Hence, it is crucial to identify biomarkers capable of accurately and specifically discriminating between AC and SCC patients [25,27], shedding light on the underlying molecular mechanisms governing the disparate phenotypes of lung cancer [26].

MicroRNAs (miRNAs) are a class of small non-coding RNAs that modulate gene expression at the post-transcriptional level through binding to the 3′ untranslated regions of target mRNAs [28]. Functionally, miRNAs play significant roles in various biological processes, including cell proliferation, differentiation, apoptosis, migration, and invasion. In the context of cancer, including lung cancer, miRNAs participate in the pathogenesis and progression as either oncogenes or tumor suppressors [3]. Consequently, miRNAs have garnered attention as potential biomarkers for cancer diagnosis, prognosis, and therapy, owing to their detectability in diverse biological fluids and tissues [3,4,5,7].

Our study aimed to characterize differentially expressed microRNAs (DEmiRNAs) between adenocarcinoma (AC) and squamous cell carcinoma (SCC) and to establish a miRNA signature that enables accurate discrimination between these two histological subtypes. Using next-generation sequencing (NGS) analysis of tumor tissue samples obtained from AC and SCC cases, we identified a total of 31 miRNAs that exhibited significant differential expression. The filtering of up and downregulated miRNAs was based on fold changes and FDR *p*-values (false discovery rate). The differentially expressed miRNAs included the following: hsa-miR-944, hsa-miR-205-5p, hsa-miR-383-5p, hsa-miR-3927-3p, hsa-miR-448, hsa-miR-3617-5p, hsa-miR-1911-5p, hsa-miR-1224-5p, hsa-miR-205-3p, hsa-miR-6510-3p, hsa-miR-3929, hsa-miR-4664-3p, hsa-miR-7974, hsa-miR-6515-3p, hsa-miR-6765-3p, hsa-miR-4709-5p, hsa-miR-3616-5p, hsa-miR-6512-5p, hsa-miR-5094, hsa-miR-6499-5p, hsa-miR-1248, hsa-miR-3128, hsa-miR-149-3p, hsa-miR-6814-5p, hsa-miR-1305, hsa-miR-597-3p, hsa-miR-3609, hsa-miR-1294, hsa-miR-5579-3p, hsa-miR-4636, and hsa-miR-3200-3p.

To enhance the diagnostic accuracy between adenocarcinoma (AC) and squamous cell carcinoma (SCC), we constructed a miRNA signature comprising 17 miRNAs based on multistep analyses including LASSO/elastic-net regression. The signature included several upregulated miRNAs, namely hsa-miR-326, hsa-miR-450a-5p, hsa-miR-1287-5p, hsa-miR-556-5p, hsa-miR-542-3p, hsa-miR-30b-5p, hsa-miR-4728-3p, hsa-miR-450a-1-3p, hsa-miR-375, hsa-miR-147b, hsa-miR-7705, and hsa-miR-653-3p. Conversely, the signature also encompassed downregulated miRNAs, specifically hsa-miR-944, hsa-miR-205-5p, hsa-miR-205-3p, hsa-miR-149-5p, and hsa-miR-6510-3p.

The initial set of DEmiRNAs significantly contrasts with the subsequent miRNA profile, comprising 31 miRNAs in the former and 17 miRNAs in the latter. Merely four downregulated miRNAs (hsa-miR-944, hsa-miR-205-5p, hsa-miR-205-3p, hsa-miR-6510-3p) are shared between the two sets. These dissimilarities can be ascribed to discrepancies in the employed methodologies and statistical analyses. The second approach, encompassing LASSO/elastic-net regression and correlation examinations, facilitates a more extensive analysis, potentially capturing miRNAs that might have been overlooked by the simpler log fold change and FDR analysis employed in the initial approach [29,30]. We believe that this expanded methodology allows for the precise identification of miRNAs with distinctive associations and plausible regulatory functions in distinguishing AC from SCC.

In our study, we evaluated a 17-miRNA signature’s discriminative capability for distinguishing between adenocarcinoma (AC) and squamous cell carcinoma (SCC) subtypes in lung cancer. To assess its performance, we generated a receiver operating characteristic (ROC) curve, resulting in an impressive area under the curve (AUC) value of 0.994. This high AUC value underscores the exceptional diagnostic performance of the miRNA signature as a stratifying biomarker. Integrating this signature into existing diagnostic algorithms has the potential to significantly enhance the accuracy and efficiency of AC and SCC subtype classification. This improvement holds promise for guiding more personalized treatment strategies and ultimately improving patient outcomes.

The miRNAs identified within our signature have been previously implicated in the pathogenesis of lung cancer. Notably, hsa-miR-326, hsa-miR-375, hsa-miR-944, and hsa-miR-205-5p have shown significant discriminatory potential for NSCLC subtyping (AC vs. SCC) based on experimental studies and data obtained from the Cancer Genome Atlas (TCGA) project [31,32]. The subtyping value of these miRNAs may be connected to their biological functions.

Specifically, hsa-miR-205, which has been recognized as either a tumor suppressor or an oncogene depending on tumor context [33], has exhibited differential expression in NSCLC subtypes [6]. Our study further supports this observation by revealing the downregulation of hsa-miR-205 in AC compared to SCC, providing additional evidence of its potential as a discriminatory marker in lung cancer. Likewise, our investigation identified hsa-miR-944 as downregulated in AC relative to SCC. Hsa-miR-944 has been associated with tumor suppressive functions and has demonstrated altered expression in several cancer types, including lung cancer [34,35]. Our findings align with existing evidence and underscore the potential significance of hsa-miR-944 as a relevant biomarker for discriminating between AC and SCC in lung cancer.

Conversely, within our signature, several miRNAs were found to be upregulated, including hsa-miR-326 [36] and hsa-miR-375 [32], both of which have been previously associated with the development and progression of lung cancer. Notably, hsa-miR-375 has exhibited a dual role as either a tumor suppressor or an oncogene, depending on the cellular context [37]. Additionally, hsa-miR-375 was shown to discriminate between adenocarcinoma (AC) and squamous cell carcinoma (SCC) with an impressive accuracy of 96% [32]. These miRNAs have been implicated in the regulation of crucial oncogenic pathways and have shown promise as prognostic markers in patients with lung cancer [36,38,39,40,41]. The incorporation of hsa-miR-326 and hsa-miR-375 into our signature strengthens its potential diagnostic utility and underscores the significance of these specific miRNAs in discriminating between AC and SCC. Their involvement suggests their active role in the underlying mechanisms and pathways that drive the differentiation between these two lung cancer subtypes. As such, these findings highlight the importance of considering these miRNAs as valuable components for developing diagnostic tools and further emphasize their potential clinical relevance in the classification of lung adenocarcinoma and squamous cell carcinoma.

However, it is important to acknowledge certain limitations in our study that deserve attention. Firstly, although our study group encompassed an adequate sample size for the initial identification and validation of differentially expressed miRNAs, expanding the cohort to include a larger population would be beneficial in further validating the diagnostic potential of the miRNA signature. Additionally, conducting functional studies to investigate the mechanistic role of these miRNAs in lung cancer subtypes would provide deeper insights into their biological significance and potential therapeutic implications.

Our highly precise miRNA signature stands as a pivotal stride towards shaping rational therapeutic strategies and guiding endeavors in drug discovery for NSCLC. Therefore, the miRNA signature can expedite the identification of the most appropriate treatment modality—a matter of paramount significance, particularly given the current focus on preoperative targeted interventions (neoadjuvant therapies). These therapeutic regimens encompass the administration of pharmacological agents prior to surgery, with the primary objective of diminishing tumor dimensions and facilitating subsequent excision, ultimately leading to the attainment of the anticipated therapeutic benefits. These therapies can be employed either in isolation or in conjunction with conventional chemotherapy or cutting-edge immunotherapy techniques. Clinical trials presently underway are diligently investigating the efficacy and safety of these interventions across diverse NSCLC histological classifications and molecular profiles [42,43,44]. Significant trials in this domain include the NEOSTAR trial, evaluating the preoperative application of nivolumab combined with ipilimumab or nivolumab monotherapy versus conventional chemotherapy in surgically treatable stage I–IIIA NSCLC patients [42]. Furthermore, the LCMC3 trial assesses the neoadjuvant use of atezolizumab combined with carboplatin/nab-paclitaxel in contrast to carboplatin/nab-paclitaxel alone in surgically treatable stage II–IIIA squamous NSCLC patients [43]. Lastly, the NADIM trial is in progress, directly comparing the preoperative application of durvalumab in conjunction with platinum-based chemotherapy versus platinum-based chemotherapy alone in surgically treatable stage IIIA NSCLC patients [44].

Clearly, the integration of miRNA signatures with histological assessment stands poised to substantially enhance the accuracy of NSCLC subtyping, thereby not only expanding the horizon for precision medicine but also paving the way for the exploration of novel therapeutic avenues targeting NSCLC.

In conclusion, our study successfully identified a panel of 17 differentially expressed miRNAs that demonstrate accurate discrimination between adenocarcinoma (AC) and squamous cell carcinoma (SCC) in lung cancer patients. This marks the initial stride towards precision medicine and the testing of novel molecularly targeted drugs. The miRNA signature exhibited excellent diagnostic performance, as evidenced by the high area under the curve (AUC) value obtained. These findings underscore the potential of miRNAs as non-invasive biomarkers for precise subtype classification in lung cancer, thereby informing treatment decisions and potentially improving patient outcomes. Further validation and translation of this miRNA signature in clinical settings are warranted to establish its utility in routine practice.

## 4. Materials and Methods

This study was conducted as part of the Polish project named “Development of Personalized Diagnostic Approaches for Malignant Neoplasms based on tumor heterogeneity and integrated genomic, transcriptomic, metabolomic, and imaging PET/MRI analysis. Preparing for Individualized Treatment” [45,46]. Written informed consent was acquired from all participants prior to the procurement of samples and the processing of clinicopathological data. The study protocol was assessed and endorsed by the Bioethics Committee of the Medical University of Bialystok, with ethical approval code R-I-002/357/2014.

### 4.1. Patients and Samples

A total of 59 surgically resected cases of non-small cell lung cancer (NSCLC) were included in the study. Among them, 31 individuals (52.5%) were histologically diagnosed with squamous cell carcinoma (SCC), while 28 individuals (47.5%) had adenocarcinoma (AC). The participants had a mean age of 65.64 years (SD = 6.95), with a median age of 65 and an age range of 49 to 77 years. Among the total participants, 23 individuals (39%) were female, while 36 individuals (61%) were male. Tumor staging distribution among the participants was as follows: 10 individuals (17%) had stage IA, 15 individuals (25.4%) had stage IB, 10 individuals (17%) had stage IIA, 9 individuals (15.2%) had stage IIB, 13 individuals (22%) had stage IIIA, and 2 individuals (3.4%) had stage IIIB. No neoadjuvant chemotherapy was administered prior to the surgical procedures. The comprehensive clinico-pathological characteristics of patients are presented in Table 3.

### 4.2. Histopathological Diagnosis

All tumor samples included in the analysis underwent histopathological evaluation. The assessment of histopathological diagnosis followed the latest WHO lung cancer classification and the International Multidisciplinary Classification of Lung Adenocarcinoma IASLC/ATS/ERS guidelines. In cases where there was uncertainty, immunohistochemical staining was performed to determine the expression of specific markers for adenocarcinoma (thyroid transcription factor-1, TTF-1) and squamous cell carcinoma (p63). This additional evaluation helped confirm the histopathological subtype of each tumor sample. Furthermore, the percentage of cancer cells present in each tumor sample was determined to ensure sufficient RNA content for subsequent isolation and analysis.

### 4.3. RNA Isolation and Quality Control

For the extraction of total RNA, including the small RNA fraction, from fresh frozen tumor samples, we employed a commercial RNA isolation kit, specifically the mirVana™ miRNA Isolation Kit (Ambion, Naugatuck, CT, USA), following the manufacturer’s protocol. Subsequently, we conducted a qualitative and quantitative assessment of the extracted RNA. The assessment was performed using spectrophotometric techniques on a NanoDrop 2000c instrument (Thermo Scientific, Waltham, MA, USA). Additionally, the concentration of the RNA solutions was determined using a fluorimetric technique with Qubit (Thermo Scientific, USA). To ensure the quality of the extracted RNA, we also evaluated the RNA integrity factor (RIN) using a microcapillary electrophoresis technique on a Bioanalyzer 2100 (Agilent Technologies, Santa Clara, CA, USA). This assessment allowed us to determine the integrity and overall quality of the RNA samples, providing confidence in their suitability for downstream analysis. By employing these rigorous quality control measures, we ensured that the extracted RNA met the necessary standards for further molecular analysis and subsequent miRNA profiling.

### 4.4. Next Generation Sequencing Analysis

For the preparation of cDNA libraries using the template of small RNA molecules, we utilized a commercially available kit, specifically the NEXTflex Small RNA Sequencing Kit v3 (gel-free and low input options) (BioScientific, Avondale, AZ, USA). This kit is designed to be compatible with Illumina technology, which is widely used for next-generation sequencing (NGS). To assess the structure and distribution of individual library fractions representing specific molecules within the smallRNA pool, we employed the microcapillary electrophoresis technique using High Sensitivity DNA chips on the Bioanalyzer 2100 system from Agilent Technologies (USA). For the selection of cDNA products corresponding to the miRNA fraction, size selection was carried out using an agarose gel electrophoresis technique. Special gel cassettes on the Blue Pippin system from Sage Science (Beverly, MA, USA) were utilized for this purpose. Following fractionation, the concentration of the cDNA libraries was determined using the KAPA Library Quantification Kit for Illumina Platforms from Roche (South San Francisco, CA, USA). Finally, the prepared libraries were subjected to sequencing on the HiSeq 4000 platform from Illumina (San Diego, CA, USA).

### 4.5. Bioinformatic Analyses

Bioinformatic analyses were conducted using the R language version 3.4.1 and environment for statistical computing, with the utilization of R-related Bioconductor module version 3.5. The sequencing reads obtained from the HiSeq 4000 instrument (Illumina) underwent base calling using the base-calling software provided by the instrument manufacturer. The reads were then subjected to overall quality control for each sample using the modular tool MultiQC v 1.7, including a number of unique and duplicate reads, content of GC pairs, sequence length distribution, sequence duplication levels, adapter content, and sequence quality scores. The reads were then trimmed to remove the adapter contamination and four random bases at both ends of the reads. Again, reads were evaluated for quality using the modular tool MultiQC v 1.7.

Subsequently, the reads were aligned against the *Homo sapiens* reference genome (Ensembl GRCh38 release) using STAR version 2.5.1b, employing the 2-pass alignment mode. The mirBase annotation was used for both mapping and read counting. After alignment, the reads were associated with known miRNAs, and the number of reads aligned within each miRNA was counted. Mapping percentages varied between the samples. The data were then normalized to remove variation between samples caused by non-biological reasons and to make the values comparable across the sample set. The counts were normalized using the TMM normalization method. For statistical testing, the data were further log transformed using the voom approach.

Quality control measures were implemented to assess the correlation between replicates and identify potential outliers. Various methods were employed for quality control, including visualization of the expression value distribution across the sample set, calculation of minimum, median, mean, and maximum expression values of the normalized samples, calculation of correlation values between samples, hierarchical clustering to group samples based on similarity, and analysis of sample relations using principal component analysis (PCA).

Following preprocessing, statistical testing was conducted to compare the sample groups of adenocarcinoma (AC) and squamous cell carcinoma (SCC). The obtained results from the testing were employed to identify differentially expressed miRNAs (DE miRNAs). Filtering of the measured miRNAs was performed based on both statistical significance and the magnitude of the difference in mean expression levels between the sample groups. Fold changes and *p*-values, which were calculated during statistical testing, served as the criteria for filtering. Specifically, a linear modeling process with the Limma package version 3.32.0 was employed for fold change (FC) analysis. The *p*-values utilized for the filtering step were the modified *t*-test *p*-values or false discovery rate (FDR). This filtering procedure aimed to identify the miRNAs that displayed the most compelling evidence of differential expression between the compared groups.

The prediction of miRNA signature from miRNA-seq data involved data normalization, transformation, and distributional checks. Moreover, it included differential abundance analysis with LASSO/elastic-net regression, considering experimental batch correction. The data were log2 transformed, and a preliminary MDS plot was generated. Significance was determined using a false discovery rate (FDR) threshold of ≤0.05.

The diagnostic utility of the identified miRNA signature was evaluated by constructing a receiver operating characteristic (ROC) curve. The ROC curve was generated by plotting the true positive rate (TPR) against the false positive rate (FPR) at different threshold settings. The MetaseqR Bioconductor package version 1.16.0 was employed for ROC curve generation.

## 5. Conclusions

Our study provides valuable insights into the potential of miRNAs as discriminatory biomarkers between adenocarcinoma (AC) and squamous cell carcinoma (SCC) subtypes in lung cancer. In the first step, we identified 31 miRNAs with significant differential expression between AC and SCC cases. Subsequently, constructing the 17-miRNA signature, we demonstrated exceptional diagnostic performance, evident by a high area under the curve (AUC) value of 0.994, highlighting its significance for precise subtype classification in lung cancer. The miRNAs, specifically hsa-miR-326, hsa-miR-375, hsa-miR-944, and hsa-miR-205-5p, identified within our signature have been previously associated with lung cancer pathogenesis and progression, further supporting their value as putative biomarkers for distinguishing between NSCLC subtypes. The integration of our miRNA signature with histological assessment holds the potential to drive more informed treatment choices, thereby enhancing patient outcomes and representing a pivotal advance towards precision medicine and the exploration of novel NSCLC-targeting drugs. However, it is crucial to emphasize that further validation and prospective trials are necessary to confirm the clinical utility of our 17-miRNA signature, as well as to ensure its capability to complement routine histological evaluation in lung cancer classification, thereby enhancing accuracy in distinguishing between adenocarcinoma (AC) and squamous cell carcinoma (SCC) subtypes.

## Figures and Tables

**Table 1:** List of identified miRNAs showing differential expression between AC and SCC groups. Legend: Average ranking value based on both *p* value and log fold change; value 1 is the strongest DE feature; upregulated miRNAs—indicated in gray; downregulated miRNAs—not indicated.

ID	logFC	FDR adj.P.Val	avgRank	Description
hsa-miR-944	−2.50024399226404	0.99998120935031	1	down
hsa-miR-205-5p	−2.56409590009272	0.99998120935031	2	down
hsa-miR-383-5p	−1.76096646669423	0.99998120935031	3	down
hsa-miR-3927-3p	−1.57116270312205	0.99998120935031	4	down
hsa-miR-448	−1.8650361500274	0.99998120935031	5	down
hsa-miR-3617-5p	1.58042497541289	0.99998120935031	6	up
hsa-miR-1911-5p	−1.86824448648207	0.99998120935031	7	down
hsa-miR-1224-5p	−1.37356958953855	0.99998120935031	8	down
hsa-miR-205-3p	−2.01278722790899	0.99998120935031	9	down
hsa-miR-6510-3p	−1.72338907430046	0.99998120935031	10	down
hsa-miR-3929	−1.28741708585196	0.99998120935031	11	down
hsa-miR-4664-3p	−1.32276408748148	0.99998120935031	12	down
hsa-miR-7974	−1.40692830818463	0.99998120935031	13	down
hsa-miR-6515-3p	−1.27296318882511	0.99998120935031	14	down
hsa-miR-6765-3p	−1.29009752267163	0.99998120935031	15	down
hsa-miR-4709-5p	1.18615415483486	0.99998120935031	16	up
hsa-miR-3616-5p	−1.34533033666886	0.99998120935031	17	down
hsa-miR-6512-5p	−1.21523532625748	0.99998120935031	18	down
hsa-miR-5094	−1.2395769248493	0.99998120935031	19	down
hsa-miR-6499-5p	−1.33109054696209	0.99998120935031	20	down
hsa-miR-1248	−1.16819512153751	0.99998120935031	23	down
hsa-miR-3128	−1.18019684918627	0.99998120935031	24	down
hsa-miR-149-3p	−1.17066684727337	0.99998120935031	25	down
hsa-miR-6814-5p	−1.03918923933257	0.99998120935031	27	down
hsa-miR-1305	−1.18333424370583	0.99998120935031	28	down
hsa-miR-597-3p	−1.09652869609583	0.99998120935031	30	down
hsa-miR-3609	−1.13169522785434	0.99998120935031	32	down
hsa-miR-1294	1.05850886870655	0.99998120935031	35	up
hsa-miR-5579-3p	−1.0917337298003	0.99998120935031	37	down
hsa-miR-4636	1.01236329971682	0.99998120935031	39	up
hsa-miR-3200-3p	−1.03714338280713	0.99998120935031	41	down

**Table 2:** List of 17 miRNAs that created a signature to differentiate AC from SCC.

qlogFC	AveExpr	t	*p*-Value	adj.P.Val	B	Name	Direction
1.29	4.84	4.57	4.55 × 10^−5^	0.0051	1.73	hsa-miR-326	up
1.14	7.40	4.49	5.89 × 10^−5^	0.0055	1.48	hsa-miR-450a-5p	up
1.33	2.04	4.40	7.70 × 10^−5^	0.0057	1.23	hsa-miR-1287-5p	up
1.83	1.47	4.36	8.73 × 10^−5^	0.0057	1.11	hsa-miR-556-5p	up
1.26	6.56	4.19	0.00015	0.0081	0.59	hsa-miR-542-3p	up
1.01	10.95	4.17	0.000158	0.0081	0.54	hsa-miR-30b-5p	up
1.08	0.036	4.00	0.000265	0.0124	0.045	hsa-miR-4728-3p	up
1.24	1.07	3.95	0.0003	0.0131	−0.082	hsa-miR-450a-1-3p	up
2.61	9.06	3.57	0.00093	0.0314	−1.15	hsa-miR-375	up
1.89	2.22	3.57	0.000947	0.0314	−1.16	hsa-miR-147b	up
1.14	2.00	3.47	0.0012	0.0378	−1.42	hsa-miR-7705	up
1.51	2.52	3.46	0.00127	0.0378	−1.44	hsa-miR-653-3p	up
−5.49	3.80	−9.72	3.81 × 10^−12^	1.61 × 10^−9^	17.59	hsa-miR-944	down
−6.19	10.90	−9.59	5.72 × 10^−12^	1.61 × 10^−9^	17.20	hsa-miR-205-5p	down
−5.60	0.3	−8.37	2.30 × 10^−10^	4.33 × 10^−8^	13.60	hsa-miR-205-3p	down
−2.29	6.45	−5.18	6.41 × 10^−6^	0.0009	3.63	hsa-miR-149-5p	down
−3.34	1.42	−4.35	9.06 × 10^−5^	0.0057	1.07	hsa-miR-6510-3p	down

**Table 3:** Patient’s characteristics. Legend: * SD—Standard deviation.

Characteristics		*n* = 59
Age (years)	Mean ± SD *	65.64 ± 6.95
	Median	65
	Range	49–77
Sex	Female	23 (39%)
	Male	36 (61%)
Tumor stage	IA	10 (17%)
	IB	15 (25.4%)
	IIA	10 (17%)
	IIB	9 (15.2%)
	IIIA	13 (22%)
	IIIB	2 (3.4%)
Histology	SCC	31 (52.5%)
	AC	28 (47.5%)

## Data Availability

The datasets analyzed during the current study are available from the corresponding author on reasonable request.
